# Demonstration of Temperature Dependent Energy Migration in Dual-Mode YVO_4_: Ho^3+^/Yb^3+^ Nanocrystals for Low Temperature Thermometry

**DOI:** 10.1038/srep36342

**Published:** 2016-11-02

**Authors:** Manoj Kumar Mahata, Tristan Koppe, Kaushal Kumar, Hans Hofsäss, Ulrich Vetter

**Affiliations:** 1 Second Institute of Physics, University of Göttingen, Friedrich-Hund-Platz 1, 37077 Göttingen, Germany; 2 Optical Materials & Bio-imaging Research Laboratory, Department of Applied Physics, Indian Institute of Technology (Indian School of Mines), Dhanbad, India

## Abstract

A dual mode rare-earth based vanadate material (YVO_4_: Ho^3+^/Yb^3+^), prepared through ethylene glycol assisted hydrothermal method, demonstrating both downconversion and upconversion, along with systematic investigation of the luminescence spectroscopy within 12–300 K is presented herein. The energy transfer processes have been explored via steady-state and time-resolved spectroscopic measurements and explained in terms of rate equation description and temporal evolution below room temperature. The maximum time for energy migration from host to rare earth (Ho^3+^) increases (0.157 μs to 0.514 μs) with the material’s temperature decreasing from 300 K to 12 K. The mechanism responsible for variation of the transients’ character is discussed through thermalization and non-radiative transitions in the system. More significantly, the temperature of the nanocrystals was determined using not only the thermally equilibrated radiative intra-4f transitions of Ho^3+^ but also the decay time and rise time of vanadate and Ho^3+^ energy levels. Our studies show that the material is highly suitable for temperature sensing below room temperature. The maximum relative sensor sensitivity using the rise time of Ho^3+^ energy level (^5^F_4_/^5^S_2_) is 1.35% K^−1^, which is the highest among the known sensitivities for luminescence based thermal probes.

The rare earth (RE) doped nanomaterials have attracted a great deal of attention owing to their narrow emission lines, large Stokes shift and longer lifetime, especially the frequency conversion ability, including down-conversion (DC) and upconversion (UC)^1^,^2^,^3^. These materials feature a broad range of potential applications e.g. display devices, optoelectronics, optical sensing, laser cooling, luminescence solar cell concentrators, bio-imaging and security applications^4^,^5^,^6^,^7^,^8^. The optical transitions in lanthanide doped nanomaterials involve the 4f orbitals, which are shielded by the 5 s and 5 p outer orbitals and thus give unique optical properties. However due to the low absorption cross-section, direct excitation of the RE ions’ 4f level results in low luminescence efficiency. The efficiency can be enhanced through sensitization processes, most commonly either through energy transfer or through charge transfer band^9^,^10^. Both processes are highly sensitive to the host matrix and therefore inspire researchers in developing suitable hosts for next generation optical sensing and display devices.

The energy transfer process from the vanadate group (VO_4_^3−^) to the RE ion has fundamental as well as technological significance^11^. The VO_4_^3−^ in YVO_4_ can be excited by near UV sources with efficient energy transfer to the RE ions. Other similar groups, except VO_4_^3−^, which have similar structural and chemical properties like PO_4_^3−^ do not show efficient energy transfer to the rare earth ions. Bourdeaux *et al*.^12^ theoretically showed that the energy transfer efficiency of the VO_4_^3−^ group is high, because the lowest electronic excited level (7A_1_) of VO_4_^3−^ is metastable and has a charge distribution that extends far into the crystal. Any excitation above this level relaxes to the 7A_1_ level which is metastable and thereby allowing energy transfer by exchange processes. For low concentrations of REs, the VO_4_^3−^ → RE energy transfer is more or less independent on the Yttrium ion, though host luminescence of YVO_4_ depends on this ion. The energy transfer from the VO_4_^3−^ group depends on the particular RE ion substituted for Yttrium^13^,^14^,^15^. Additionally, it is well known that YVO_4_ has low optical phonon energy (880 cm^−1^) and this feature introduces the possibility for frequency upconversion, which is defined as a non-linear optical process, converting low energy photons into high energy photons. Usually, in upconversion process near infrared (NIR) light is converted into ultra-violet (UV) or visible light. The ability of converting UV to visible (DC) as well as NIR to visible (UC) by the same material makes it a promising one for novel applications, including efficiency enhancement of solar cells, upconversion lasers, bio-imaging, fluorescence labeling, etc^9^. Therefore yttrium vanadate has been chosen as a host material for rare earth doping in this study.

Herein, the aim was to explore the host to rare earth energy transfer through time-resolved spectroscopy at different temperatures in this dual mode material which exhibits DC and UC emissions. Also, the question which we have tried to answer is how fast the energy migrates to Ho^3+^ ions from the vanadate groups of the host. We have chosen the Ho^3+^/Yb^3+^ conjugate for this study as they produce strong green UC emission in some other host materials and give green DC emission as well^16^,^17^. The Yb^3+^ ions were co-doped to sensitize the Ho^3+^ ions upon NIR excitation due to large absorption cross-section of Yb^3+^ ions at 980 nm. The nanoparticles were prepared under hydrothermal treatment using ethylene glycol as a chelating agent to prevent the growth of the particles during synthesis process. The investigation performed in the current framework shows the importance of this material not only as a dual mode luminescent material but also as an excellent candidate for optical thermometry. Accurate temperature sensing in a non-invasive way is one of the most interesting and demanding applications because of its superiority over conventional contact based thermometers^18^,^19^,^20^. In general, the temperature sensing of rare earth doped materials is determined by two commonly used methods i. e. decay time method and fluorescence intensity ratio (FIR) technique. Since the past decade, temperature sensing has been studied using luminescent micro-crystalline compounds at the tip of scanning thermal probes, at the tips of optical fibers or on silica-on-silicon waveguides^21^,^22^,^23^ which seem to be unsuitable because microcrystals at the tip entail a drawback in that the material may act as a thermal insulator. Apart from this, due to light scattering by rough surfaces of larger particles, the resolution of temperature sensing is also reduced. In this paper, the temperature sensing performance has been determined through fluorescence intensity ratio, decay time and rise time methods. The FIR method is widely followed and studied in many previous works^24^,^25^,^26^,^27^,^28^. But less work is available in literature since the first promising report was published on temperature sensing considering the rise time and to the best of our knowledge, there are only three reports till today^29^,^30^,^31^. All these reports are based on europium ions in Y_2_O_3_, SrY_2_O_4_, and BaY_2_ZnO_5_ hosts. Lojpur *et al*.^29^ and Ranson *et al*.^30^ have shown the variation of the rise time of transients, which exhibit temperature sensing behavior through temperature dependence of the rise time. Rise time of the ^5^D_0_ level of Eu^3+^ in Y_2_O_3_ host was investigated by Ranson *et al*. through monitoring the 611 nm emission with the assumption that Eu^3+^ exists at two lattice sites in the Y_2_O_3_ host. They concluded that the increase in rise time is due to energy transfer into the ^5^D_0_ level of Eu^3+^ at C_2_ sites from the ^5^D_1_ level of Eu^3+^ at C_2_ and C_3i_ sites. The YVO_4_: Ho^3+^/Yb^3+^ has shown very high sensitivity at low temperature using the FIR technique and decreases fast as the temperature of the material increases. On the other hand, the rise time method shows high sensitivity above 150 K with a maximum of 1.35% K^−1^ at room temperature (RT). The decay time of the host emitting level (vanadate) has also been exploited in temperature sensing purpose and gives the maximum sensitivity of 1.22% K^−1^ at room temperature. The variation in intensity of rare earth emission as well as host emission reflects that the sensitization of rare earth by the host is a temperature dependent process. Therefore, in this work we have investigated YVO_4_: Ho^3+^/Yb^3+^ nanocrystals in order to understand the energy migration through time-resolved spectroscopy and explore the feasibility of temperature sensing within the temperature range 12 K to 300 K.

## Results and Discussion

### Crystal structure and particle size

**Figure 1:**
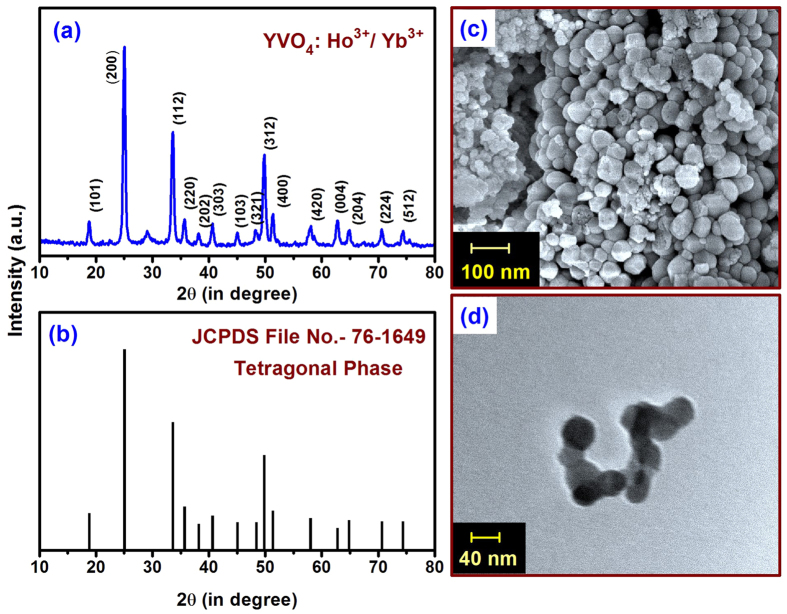
(**a**) X-ray diffraction pattern of YVO_4_: Ho^3+^/Yb^3+^ powder prepared at 1073 K. (**b**) Standard JCPDS data (card No. 76-1649) of YVO_4_. (**c**) Field-emission scanning electron microscopy image. (**d**) Transmission electron microscopy image of as-synthesized YVO_4_: Ho^3+^/Yb^3+^ particles.

Figure 1a shows the XRD pattern of the synthesized YVO_4_: Ho^3+^/Yb^3+^ nanoparticles. It was observed that the diffraction peaks of the sample coincide with standard data on YVO_4_ (JCPDS No. 76-1649, Fig. 1b) and no extra peak for impurity is seen, which indicates that the dopants Ho^3+^ and Yb^3+^ enter uniformly into the lattice of YVO_4_. The crystalline structure is tetragonal zircon type with space group D_4h_. In addition, the relatively intense reflection peaks suggest that the nanoparticles are highly crystalline in nature. The YVO_4_ crystallizes with zircon type structure, when V^5+^ ion in the [VO_4_]^3−^ groups are coordinated tetrahedrally with O^2−^ ions and the Y^3+^ ions are located within dodecahedra of eight O^2−^ ions. The overall structure of YVO_4_ is composed of alternating edge sharing of YO_8_ and VO_4_ tetrahedra. Consequently, the substitution of Y^3+^ ions with Ho^3+^ and Yb^3+^ will result in emissions that are characteristic for D_2d_ point symmetry. The field emission scanning electron microscopy (FESEM) and transmission electron microscopy (TEM) images of the as-synthesized sample are shown in the Fig. 1c,d respectively. The TEM image was measured on a Cu-grid coated with particles dispersed in ethanol. Both the FESEM and TEM images are consistent and particles have similar dimension. The average particles size was determined to be ~40 nm.

### Steady-state photoluminescence properties: down-conversion and up-conversion

**Figure 2:**
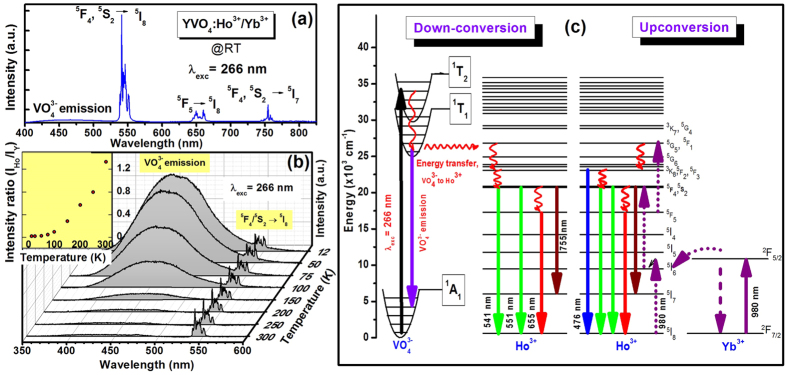
(**a**) Photoluminescence spectrum at room temperature under 266 nm laser light excitation. (**b**) Temperature dependent photoluminescence emission spectra; inset shows the intensity ratio of holmium to vanadate at different temperatures. (**c**) Schematic diagram of emission processes under 266 and 980 nm laser light excitations in YVO_4_: Ho^3+^/Yb^3+^ nanocrystals.

The down-conversion emission spectrum of YVO_4_: Ho^3+^/Yb^3+^ is shown in Fig. 2a. Upon UV (266 nm) excitation, the sample exhibits strong green (541, 551 nm) with weak red (650 nm), blue (476 nm) and NIR (755 nm) emission bands associated with ^5^F_4_/^5^S_2_ → ^5^I_8_; ^5^F_5_ → ^5^I_8_; ^5^F_3_ → ^5^I_8_ and ^5^F_4_/^5^S_2_ → ^5^I_7_ transitions respectively, of Ho^3+^ ions at the D_2d_ site of YVO_4_ (Fig. 2c). The 266 nm excitation falls in the broad absorption band of the host lattice and hence Ho^3+^ emission occurs due to energy transfer from host to RE. The emission from the VO_4_^3−^ group was also observed in 400–550 nm region which reveals that the whole amount of absorbed energy is not transferred to the Ho^3+^ ions; some of the VO_4_^3−^ groups de-excite radiatively and produce the broad emission band. In the emission process from Ho^3+^ ions, first UV light is absorbed by a vanadate group and then the excited energy is transferred to the higher lying excited states of Ho^3+^ ions. Finally the emission from holmium ions occurs in the visible and NIR regions. The emission intensity of Ho^3+^ depends on the UV-absorption by the YVO_4_ host and the efficiency of energy transfer from host to the Ho^3+^ ion. In the YVO_4_ host, the Y-bond has strong absorption of UV-light at the short wavelength whereas the V–O bond can absorb relatively longer wavelength of UV light. According to Goodenough *et al*.^32^ the super-exchange between cation-anion-cation is dependent on the angle in the chain paramagnetic ion - oxygen ion - paramagnetic ion. This interaction is strong for σ-bonding (angle ~180°) where the wavefunction overlaps and it is weak for π-bonding (angle ~90°). In the present case the V-O-Y angle is 170° (which is closer to the case of σ-bonding). Therefore, due to overlapping of V-O-Y wavefunctions it induces an efficient energy transfer from VO_4_^3−^ → Ho^3+^ 32,33,34.

The temperature dependent photoluminescence spectra are shown in Fig. 2b. The luminescence due to vanadate (VO_4_^3−^) groups transitions is very strong at low temperatures. At low temperature the non-radiative relaxation from VO_4_^3−^ decreases. Also, it is seen from Fig. 2b that at low temperature the intensity of vanadate group transition is very high compared to Ho^3+^ emission. At low temperatures most of the excited vanadate groups relax radiatively to ground state and the energy transfer to Ho^3+^ is not efficient. The VO_4_^3−^ emission intensity is strongly dependent on the temperature due to the strong electron-phonon interaction (no shielding as compared to RE^3+^ ions). It is expected that Ho^3+^ emission should also be increased in intensity as temperature lowers but prominent enhancement in Ho^3+^ emission intensity is not observed^19^.

**Figure 3:**
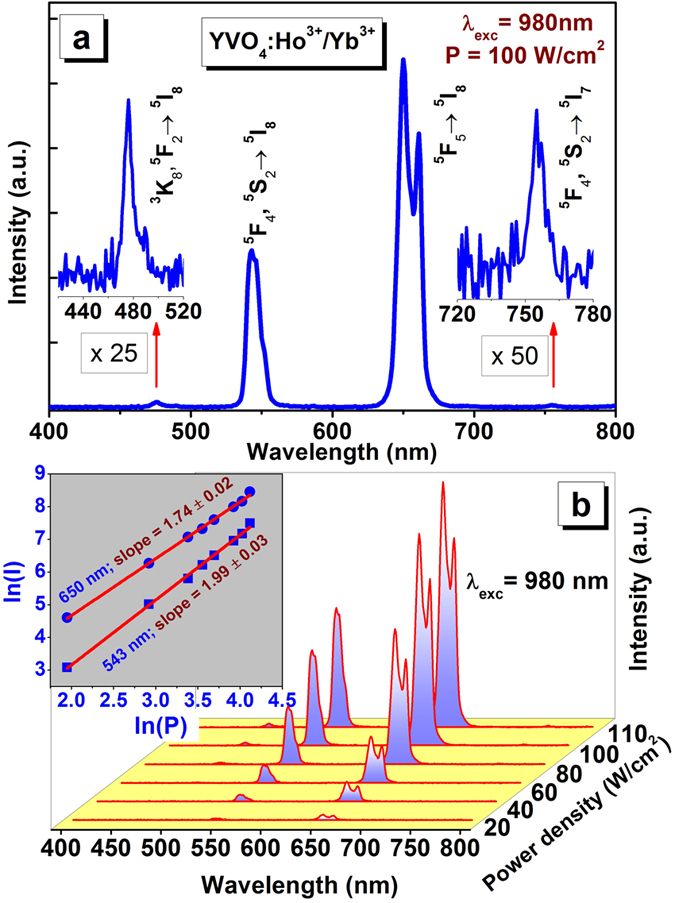
(**a**) Upconversion emission under 980 nm diode laser excitation. (**b**) Pump power dependent emission spectra; inset shows the ln(I)-ln(P) plots to calculate number of absorbed photons.

The UC emission spectrum of YVO_4_: Ho^3+^/Yb^3+^ (excited at 980 nm) at room temperature is shown in Fig. 3a. The UC emission is very intense in the red region followed by green emission. In addition, UC emission is also observed in blue and NIR regions but their intensity is rather weak, as shown in the inset of Fig. 3a. The UC emission bands are observed at 476, 543, 650 and 750 nm corresponding to ^5^F_2_, ^3^K_8_ → ^5^I_8_; ^5^F_4_, ^5^S_2_ → ^5^I_8_; ^5^F_5_ → ^5^I_8_ and ^5^F_4_, ^5^S_2_ → ^5^I_7_ transitions of Ho^3+^ ion, respectively. The Yb^3+^ ions act as sensitizer for Ho^3+^ emission at 980 nm. In the UC emission, the logarithmic plot of emission intensity vs. power density of the different bands, indicates the number of absorbed photons in a particular UC transition^35^. The excitation mechanism could also be determined with the help of pump power dependent UC study. In YVO_4_: Ho^3+^/Yb^3+^, the slope for the bands at 540 and 650 nm were observed to be 1.99 and 1.74 (inset of Fig. 3b), respectively, which confirms the absorption of two incident photons for the green and red UC emission bands.

The whole UC process involved in the Ho^3+^-Yb^3+^ system is shown schematically in Fig. 2c. The 980 nm radiation excites the Ho^3+^ and Yb^3+^ ions resonantly through ground state absorption (GSA) process. Since the absorption cross-section for Ho^3+^ ion is low, the GSA process is very weak for Ho^3+^. The Yb^3+^ ions have an absorption cross-section at 980 nm about 10 times higher than the Ho^3+^ ions. Moreover, the concentration of Yb^3+^ ions in the present material is nearly 15 times than that of the Ho^3+^ ions. Thus, most of the incident photons are absorbed by Yb^3+^ ions and transfer their energy to Ho^3+^ ions through energy transfer process. Different lower lying levels are populated via non-radiative relaxations from the upper lying levels as shown in Fig. 2c.

### Time-resolved photoluminescence properties: rate equation model, energy transfer and transients

**Figure 4:**
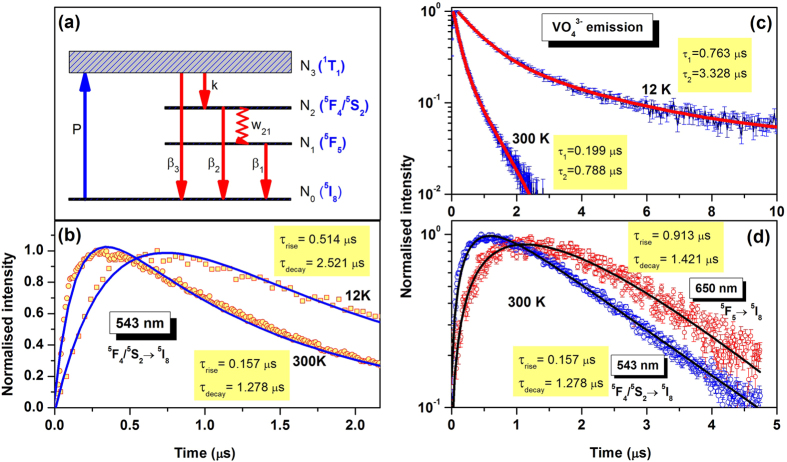
(**a**) A model representing energy transfer from host to holmium ion. (**b**) Fittings of the experimental data according to equation (7) for 543 nm emission (^5^F_4_/^5^S_2_ → ^5^I_8_) at 12 K and 300 K. (**c**) Decay times at 300 K and 12 K for vanadate emitting level. (**d**) Rise and decay times of 543 nm and 650 nm emitting levels of Ho^3+^ in YVO_4_.

The whole luminescence process upon 266 nm excitation has been simplified and the partial energy levels of Ho^3+^ and transitions are schematically shown in Fig. 4a. Here N_i_ represents the population of the energy levels and k represents the rate of energy migration from vanadate to ^5^F_4_/^5^S_2_ level of Ho^3+^ ion. β_3_ is the radiative decay rate of the vanadate emitting level. β_2_ and w_21_ are the radiative and non-radiative decay rates respectively of the ^5^F_4_/^5^S_2_ → ^5^I_8_ transition. β_1_ is the radiation decay rate of the ^5^F_5_ → ^5^I_8_ transition.

The rate equations for the excited state populations are:


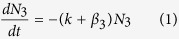


where, N_3_(t = 0) =A_0_


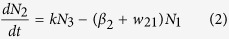



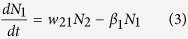


Direct excitation of the Ho^3+^ ions by 266 nm radiation has been neglected here.

Solving equation (1):


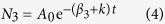


From equation (2),


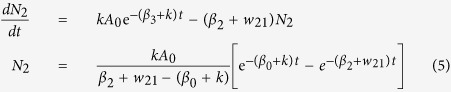


where, β_0_ is the intrinsic decay rate of N_3_.

From equation (3),


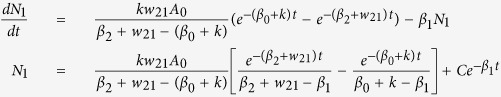


where, C is a constant given by,


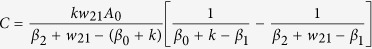


Therefore, the fluorescence intensity corresponding to decay rates β_3_, β_2_ and β_1_ are given by


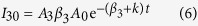



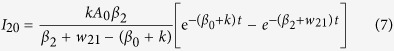



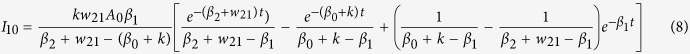


The first term in equation (7) represents the decay of the emitter level while the second term represents the increase in population of the emitter levels (^5^F_4_/^5^S_2_). The rise time and decay time are the reciprocal of the corresponding exponent values of the exponential terms. A similar solution was obtained in Ref. 36. The Ho^3+^ emission thus show an initial rise with a subsequent decay. The fittings of the transients according to equation (7) for the 543 nm emission band (^5^F_4_/^5^S_2_ → ^5^I_8_) at room temperature and 12 K are shown in Fig. 4b. The rise times at 300 K and 12 K were measured as 0.157 μs and 0.514 μs respectively while the decay times were 1.278 μs and 2.521 μs, respectively. The decay curve fittings with decay times for VO_4_^3−^ emitting level at two extreme temperatures are shown in Fig. 4c. The fittings of the red emission transient [equation (8)] at 650 nm (^5^F_5_ → ^5^I_8_) along with 543 nm band at room temperature are shown in Fig. 4d.

**Figure 5:**
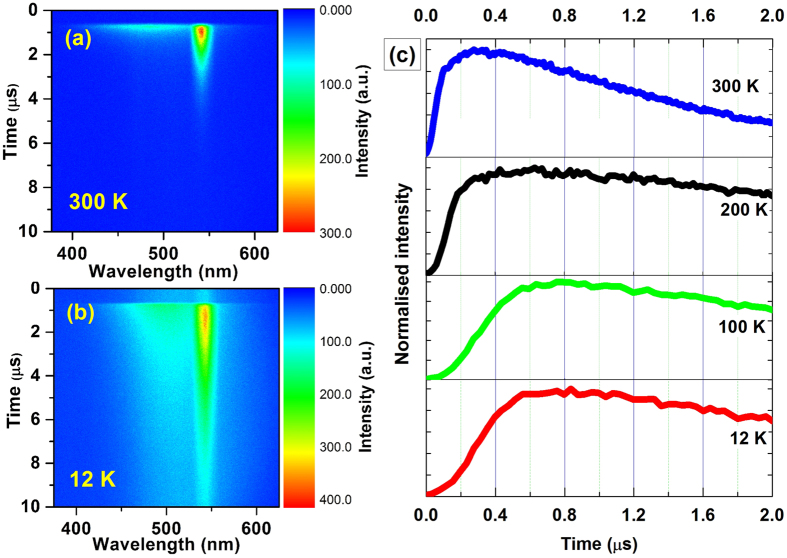
Streak camera images at (**a**) 300 K (b) 12 K (**c**) Transients of 543 nm emission (^5^F_4_/^5^S_2_ → ^5^I_8_) at different temperatures.

The streak camera images taken at 300 K and 12 K are shown in Fig. 5a,b. The luminescence transients for Ho^3+^ emission at different temperatures are shown in Fig. 5c. These curves indicate that the decay time as well as rise time increases as the temperature decreases. Shortly after the laser pulse the transients of both the emitting levels of Ho^3+^ are disturbed by the decay component of the vanadate luminescence. As the Ho^3+^ ions are excited via energy transfer from vanadate groups, the rise time indicates the upper limit for energy transfer to holmium. This value presents the upper limit only, because the ^5^F_4_/^5^S_2_ level of Ho^3+^ are not populated directly but through a relaxation process involving several higher levels of holmium. Taking the time necessary for relaxation to the ^5^F_4_/^5^S_2_ level into account, we can say that the energy transfer to holmium is even faster than 157 ns. The energy transfer between different vanadate groups is a thermally excited process and it occurs at a sufficiently high rate between neighbors. The radiationless processes become dominant with increasing temperature of the material and as a result the intensity of vanadate emission as well as lifetime of VO_4_^3−^ groups decreases. Although, all the Ho^3+^ ions are excited via VO_4_^3−^ energy transfer, the luminescence intensity of holmium is also affected. The intensity of holmium emission increases with increasing the sample temperature. We therefore conclude that the probability of energy transfer from vanadate to holmium becomes slow at low temperature though the energy transfer is faster than the radiationless processes.

Figure 4b shows the single exponent fitting of emission from ^5^F_4_/^5^S_2_ level at two different temperatures. The decay time is found to increase from 1.278 μs to 2.521 μs as the temperature is lowered from RT (300 K) to 12 K. It is assumed that 266 nm excite the vanadate groups from their ground state and there is some chances of radiative energy transfer to the Ho^3+^ ions. Figure 2c indicates concurrent feeding of the ^5^F_4_/^5^S_2_ level by upper lying levels, which is observed as a part of rise time in the transients. The energy transfer time from vanadate to 543 nm emitting level is maximum of 514 ns at 12 K. The maximum energy transfer time from vanadate to holmium becomes faster with increasing temperature and decreases from 514 ns to 127 ns. Decay times of both the VO_4_^3−^ and 543 nm emitting levels are also found to decrease with increasing sample temperature. Moreover, the temperature dependent decay time variation of the VO_4_^3−^ emitting level and rise time of 543 nm emitting level has similar trend which again reflects the energy transfer from vanadate group to holmium ion. The decay time of VO_4_^3−^ emitting level and rise time of 543 nm emitting level both show temperature sensitivity from 150 K to 300 K and below 150 K it becomes nearly invariable with temperature. However the decay time of ^5^F_4_/^5^S_2_ level also shows temperature dependent behavior, and decay time becomes nearly double at 12 K compared to that at 300 K and varies from 2.521 μs to 1.278 μs as the temperature increases from 12 K to 300 K. The decay curve of vanadate emission shows two components- short and long decay. The long decay component of decay time is consistent with previous reports in this material^37^. The long and short components of vanadate groups may be because of complex nature of excited state of vanadate group^38^. The origin of short decay time is not clear and possible reasons may include rapid quenching of the VO_4_^3−^ groups at particle surface, singulet emission of VO_4_^3−^ groups and multi-photonic process under UV-excitation^39^.

At steady-state excitation, the ratio of the emission intensities of Ho^3+^-luminescence to VO_4_^3−^ emission is given by^40^:


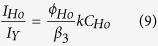


where, ϕ_Ho_ is the quantum efficiency of the Ho^3+^ emission (^5^F_4_/^5^S_2_ → ^5^I_8_), C_Ho_ (=2.48 × 10^20^/cm^3^ in present case) is the concentration of Ho^3+^-ions.

The above eq. can be written as^40^


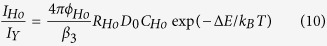


where, R_Ho_ is the trapping radius (the critical interaction distance) of Ho^3+^ ions and D_0_ is the co-efficient of excitation diffusion at T = 0 K.

The intensity ratio of vanadate to holmium emission is shown in inset of Fig. 2b. The figure shows that the intensity ratio is nearly constant below ~50 K and at higher temperatures it increases exponentially. This can be understood from equation (10) which predicts that the intensity ratio should decrease exponentially with lowering the temperature. The experimentally obtained result is consistent with equation (10) from room temperature (300 K) to ~50 K. But at low temperature (below ~50 K) the intensity ratio is constant which is due to the presence of long-range resonance energy transfer from excitons to Ho^3+^ which has not been accounted for in Fig. 4a.

**Figure 6:**
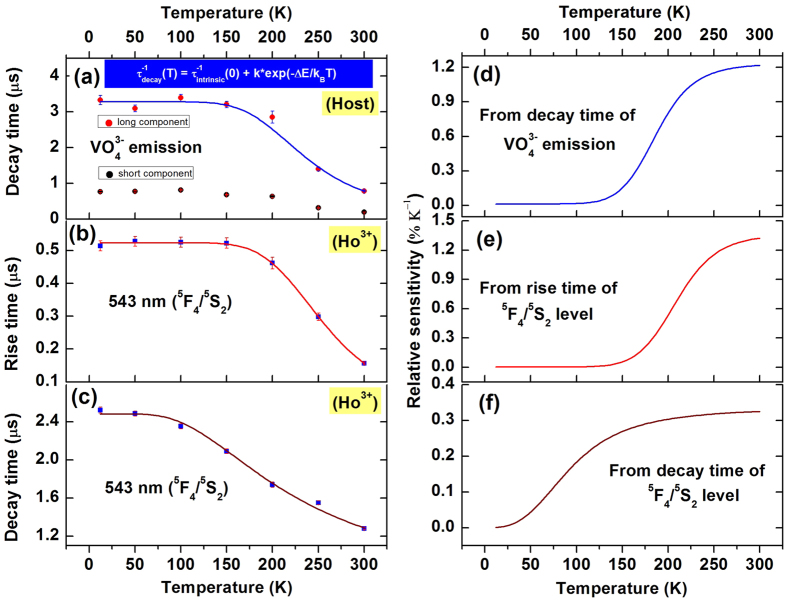
(**a**) Temperature dependence of the vanadate decay time; Temperature dependence of (**b**) Rise time (**c**) Decay time of 543 nm emitting level (^5^F_4_/^5^S_2_). Calculated approximation of relative sensor sensitivity using (**d**) Decay time of vanadate emission (**e**) Rise time of ^5^F_4_/^5^S_2_ level (f) Decay time of ^5^F_4_/^5^S_2_ level in YVO_4_: Ho^3+^/Yb^3+^.

The rise and decay times for the red emitting level (^5^F_5_) were measured as 0.913 μs and 1.421 μs, respectively. The higher rise time of ^5^F_5_ compared to ^5^F_4_/^5^S_2_ is attributed to the contribution of energy transfer from the upper lying levels ^5^F_4_/^5^S_2_. However, as there is already a feeding exists to the ^5^F_5_ level from ^5^F_4_/^5^S_2_ level, the decay time of this level may have shorter value than 1.421 μs. The evolution of the decay time of the VO_4_^3−^ emission band with temperature are shown in Fig. 6a. According to Fig. 4a, the luminescence of the host can be expressed as^37^


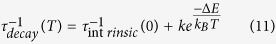


where, τ_decay_ is the observed decay time at different temperatures; τ_intrinsic_ is the intrinsic decay time of the level, k is the energy migration rate, ΔE is the energy barrier (activation energy). From the fitting of experimental data, the activation energy for migration is obtained as 1146 cm^−1^, the intrinsic decay time of the fluorescence level is 3.267 μs and the migration rate is 2.84 × 10^5^ s^−1^. However, previously the migration rate and activation energy in pure YVO_4_ was reported as 625.5 cm^−1^ and 5.0 × 10^5^ s^−1^, respectively^37^. Equation (11) can be used to explain the lifetime data shown in Fig. 6a. The lifetime data exhibit a constant value within 12 K to ~150 K and from 150 K to 300 K, it decreases continuously. The radiation-less quenching of the vanadate emission can be introduced to explain the observation above 150 K. The diffusion co-efficient D for exciton migration is determined from equation (9). Assuming the standard value of trapping distance (3.15 Å) between Ho^3+^ and nearest VO_4_^3−^ group for energy migration, the intrinsic decay time (=3.267 μs), migration rate k (=2.84 × 10^5^ s^−1^), the diffusion co-efficient is calculated as D = 2.85 × 10^−9^ cm^2^/s. The expressions for diffusion length and hopping time for energy migration are given by^40^: L^2^ = 2Dτ_intrinsic_ (0) and t_H_ = α^2^/6D, where, α is VO_4_^3−^- VO_4_^3−^ separation (4.75 Å) in YVO_4_. Using the value of D, calculated above, the above equations give the value of L = 1.4 × 10^−7^ cm and t_H_ = 1.32 × 10^−7^ s. The value of t_H_ corresponds to the energy migration rate between two vanadate groups. Now, the number of steps in the vanadate’s random walk before transferring to the Ho^3+^ ion can be calculated as n = τ_intrinsic_ (0)/t_H_ = 25 (approx.). Therefore, energy migration to holmium ions from vanadate groups takes place after 25 steps of vanadates own energy transfer and the energy migration process is dependent on temperature of the material.

### Temperature sensing

The luminescence based temperature sensing method uses the temperature-dependent luminescence properties of the material. It can overcome some limitations (e.g. hazardous environment, strong electromagnetic noise, etc.), which are not possible by traditional thermometers. Temperature dependent decay time of ^5^F_4_/^5^S_2_ level was fitted according to^41^


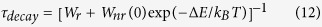


The temperature dependent rise time were also fitted to a similar type of Arrhenius equation as used in ref. 31.

The decay time of vanadate emission level (Fig. 6a) and rise time of ^5^F_4_/^5^S_2_ emission level (Fig. 6b) are nearly constant below T < 150 K. Their dependence on temperature is exhibited within 300 ≥ T ≥ 150 K. Therefore, the rise time of ^5^F_4_/^5^S_2_ emitting level and decay time of the vanadate level could be used in temperature sensing in the range of 150 to 300 K. The decay time of the ^5^F_4_ emitting level shows strong temperature dependence (Fig. 6c) in the whole range of temperature (12 to 300 K). This characteristic can also be utilized for temperature sensing in wider range. The variation of decay time with temperature can be defined according to, τ^−1^ = 0.4 + 1.6 * exp(−449/T).

**Figure 7:**
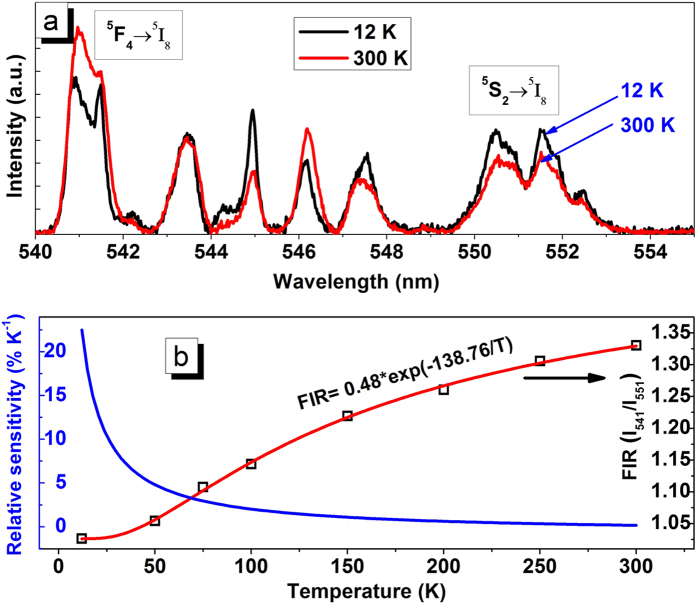
(**a**) Temperature dependent emission spectra of ^5^F_4_ and ^5^S_2_ emission bands. (**b**) Temperature dependent fluorescence intensity ratio and calculated relative sensor sensitivity.

Furthermore, we have tried to compare the decay time based temperature sensing with the FIR based temperature sensing. The temperature dependence emission spectra of 541 and 551 nm bands under 266 nm UV-excitation are shown in Fig. 7a. The figure clearly shows that the relative variation of these two bands is dependent on temperature. The emission intensity of 541 nm band increases with increasing temperature while the intensity of 551 nm band decreases with increasing temperature. In FIR based temperature sensing the emission from two closely spaced levels ^5^F_4_ and ^5^S_2_ have been used. As the temperature increases the population of the ^5^F_4_ level (541 nm emitting band) increases and the fluorescence from this level increases gradually. The FIR technique uses the intensity ratio of two separate fluorescence wavelengths emitted from two closely spaced thermally coupled levels^24^,^25^,^26^,^27^,^28^,^42^,^43^,^44^.

In this case,


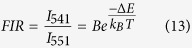


where, B is dependent on the spontaneous emission rate, frequency of emitted radiation and degeneracy of the two emitting levels^24^,^25^. The FIR involves various material-specific constants and is independent of the source intensity. It depends on temperature and increases with increasing temperature. The formula given above can be used to fit the experimental data (Fig. 7b) directly and the temperature dependent FIR can be used to calibrate the measurements. According to equation (13), the values of the parameters B and ΔE, are found to be B = 0.48 ± 0.01 and ΔE = 96.43 ± 3.93 cm^−1^ for this system. The FIR is seen to increase with temperature.

The sensor sensitivity is an important parameter and is defined as the change in signal with temperature. The relative sensitivity, S_r_ is defined as^45^


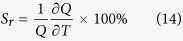


where, Q is either decay time, rise time or FIR.

The relative sensitivity of temporal thermometry is calculated and presented in Fig. 6(d–f). The maximal values of relative sensitivity using the rise time and decay time of ^5^F_4_/^5^S_2_ are 1.35 and 0.33% K^−1^ respectively at 300 K while based on decay time of vanadate emitting level, the maximum sensitivity is 1.22% K^−1^ at 300 K. According to FIR technique, the value of relative sensitivity has a maximum of 22.2% K^−1^ at 12 K and decreases with increasing temperature to 0.2% K^−1^ at 300 K. (Fig. 7b). A similar nature of temperature sensing was achieved within 150 to 300 K using Y_2_O_3_:Er^3+^/Yb^3+^ particles by Lojpur *et al*.^45^. Thus the temperature sensor developed in the present work has the highest thermal sensitivity compared to other materials^9^,^18^,^24^,^26^,^27^,^28^,^29^,^30^,^31^,^41^,^42^,^43^,^44^,^45^. If we compare the results of decay time, rise time and FIR studies of the present material then two different types of behavior of sensitivity is observed. In decay time and rise time studies the sensitivity is found to increase with temperature while in case of FIR studies sensitivity decreases rapidly with temperature. Therefore, the FIR method as well as temporal methods for optical thermometry using YVO_4_: Ho^3+^/Yb^3+^ nanocrystals could be used upon their suitable range of sensitivity though both the methods have competitive advantages to each other.

## Conclusions

In conclusion, different optical functions are observed with Ho^3+^/Yb^3+^ doping in zircon-type structure of YVO_4_. In DC, the YVO_4_: Ho^3+^/Yb^3+^ nanocrystals exhibit characteristic sharp emissions in the green region along with broad emission from the vanadate groups centered at ~480 nm. The intensity of vanadate emission is much weaker at room temperature and increases profoundly with decreasing temperature. At the same time, the holmium emission does not show increase in intensity at low temperature indicating the dependence of energy transfer to the holmium ions on thermalization and non-radiative pathways of the system. The Ho^3+^/Yb^3+^ doped YVO_4_ particles emit strong visible light with both DC and UC processes and therefore, could be used to convert the UV as well as NIR into visible light as a dual mode nanoparticle phosphor. The maximum time for energy transfer to the holmium ions from the host is measured as 157 ns at RT and it becomes slow (514 ns) at 12 K. There are several energy transfer steps within the VO_4_^3−^ groups before, transferring it to the holmium ion. The temperature sensor behavior of the material has been predicted within 12 K to 300 K using FIR method, decay time method and rise time method. The FIR method and temporal method both have different sensitivity range and thus suitable for optical thermometry. The maximum sensitivity using FIR, decay time of the vanadate emitting level, decay time of the ^5^F_4_/^5^S_2_ level of Ho^3+^ and rise time of the ^5^F_4_/^5^S_2_ levels of Ho^3+^, are 22.2% K^−1^ at 12 K, 1.22% K^−1^ at 300 K, 0.33% K^−1^ at 300 K and 1.35% K^−1^ at 300 K, respectively.

## Methods

Ho^3+^ and Yb^3+^ doped YVO_4_ was prepared through a hydrothermal route. The concentrations of Ho^3+^ and Yb^3+^ were chosen such that the maximum upconversion emission intensity under 980 nm excitation is observed. Some previous reports show that the optimized concentrations for Ho^3+^ and Yb^3+^ are about 0.2 and 3.0 mol%, respectively in several hosts^16^,^17^ for UC emission. The starting materials viz. V_2_O_5_, Y_2_O_3_, Ho_2_O_3_, and Yb_2_O_3_ were taken with 99.99% purity and their nitrates were prepared by dissolving these oxides in concentrated nitric acid. All these nitrates were mixed in a beaker with double amount of distilled water and placed on a magnetic stirrer at 80 °C for 3 h. Ethylene glycol (EG) was used as chelating agent for metal ions, keeping the molar ratio of metal ions to EG at 1:2. Ammonium hydroxide (NH_4_OH) was mixed drop wise under vigorous stirring and the pH value of the obtained solution was kept at 8.0. Then the solution of these reagents was stirred on a magnetic stirrer at 80 °C. The resulting solution was then transferred to an autoclave for hydrothermal treatment at 200 °C for 24 h. After treatment, the solution was cooled down to room temperature and the precipitate was collected by centrifugation. The as-synthesized powder was annealed at 800 °C for 3h in air.

The phase structure of the powder was characterized via X-ray diffraction pattern carried out on a Bruker-D8 Advanced X-ray diffractometer using Cu-K_α_ (1.5405 Å) radiation source over an angular range 10° ≤ 2*θ* ≤ 80°. Field emission scanning electron microscope (FESEM) images were taken using a ZEISS SUPPRA 55. Transmission electron microscope (TEM) of the prepared sample was studied using Hitachi (H-7500) 120 kV model equipped with CCD Camera. The upconversion emission spectra were recorded using 980 nm diode laser excitation on an SP2300 grating spectrometer (Princeton Instruments, USA). The temperature dependent downconversion emissions from 12 K to 300 K were measured on SPEX 1000M spectrometer under excitation of 266 nm ultra-violet (UV) light. The sample chamber was cooled down using a He-closed-cycle refrigerator at a pressure of 10^−6^ mbar. The lifetime measurements were carried out under excitation with 266 nm light emitted from a Ti-sapphire laser, Mira 900-F (Coherent) pumped by 532 nm laser (Verdi 10) with fluorescence set up consisting of a streak camera (Hamamatsu C10910), water-cooled CCD (Hamamatsu Orca R2), a synchronous delay generator (Hamamatsu C10647-01), a delay unit (Hamamatsu C1097-05) in combination with pulse picker was used to produce laser ‘pulses’.

## Additional Information

**How to cite this article**: Mahata, M. K. *et al*. Demonstration of Temperature Dependent Energy Migration in Dual-Mode YVO4: Ho^3+^/Yb^3+^ Nanocrystals for Low Temperature Thermometry. *Sci. Rep.* **6**, 36342; doi: 10.1038/srep36342 (2016).

**Publisher’s note:** Springer Nature remains neutral with regard to jurisdictional claims in published maps and institutional affiliations.

## References

[b1] Auzel F. Upconversion and Anti-Stokes Processes with f and d Ions in Solids. Chem. Rev. 104, 139–174 (2004). 14719973 10.1021/cr020357g

[b2] Wang F. & Liu X. Recent advances in the chemistry of lanthanide-doped upconversion nanocrystals. Chem. Soc. Rev. 38, 976–989 (2009). 19421576 10.1039/b809132n

[b3] Kotov N. Bioimaging: The only way is up. Nat. Mater. 10, 903–904 (2011). 22109602 10.1038/nmat3181

[b4] Downing E., Hesselink L., Ralston J. & Macfarlane R. A three-color, solid-state, three-dimensional display. Science 276, 1185–1189 (1996).

[b5] Chatterjee D. K. & Zhang Y. Upconverting nanoparticles as nanotransducers for photodynamic therapy in cancer cells. Nanomedicine 3, 73–82 (2008). 18393642 10.2217/17435889.3.1.73

[b6] Liu Q., Feng W., Yang T., Yi T. & F. Li, Upconversion luminescence imaging of cells and small animals. Nat. Protoc. 8, 2033–2044 (2013). 24071909 10.1038/nprot.2013.114

[b7] Fischer S. . S. W. Enhancement of silicon solar cell efficiency by upconversion: Optical and electrical characterization. J. Appl. Phys. 108, 044912 (2010).

[b8] Chen J. & Zhao J. X. Upconversion Nanomaterials: Synthesis, Mechanism, and Applications in Sensing. Sensors 12, 2414–2435 (2012). 22736958 10.3390/s120302414 PMC3376553

[b9] Singh A. K., Singh S. K., Gupta B. K., Prakash R. & Rai S. B. Probing a highly efficient dual mode: down–upconversion luminescence and temperature sensing performance of rare-earth oxide phosphors. Dalton Trans. 42, 1065–1072 (2013). 23114691 10.1039/c2dt32054a

[b10] Huang F. . Origin of near to middle infrared luminescence and energy transfer process of Er^3+^/Yb^3+^ co-doped fluorotellurite glasses under different excitations. Sci. Rep. 5, 8233 (1–6) (2015). 10.1038/srep08233 PMC5389029 25648651

[b11] Blasse G. Energy transfer in oxidic phosphors. Philips Res. Rept. 24, 131–144 (1969).

[b12] Boudreaux D. S. & La France T. S. Energy transfer from the excited VO^4^_3_− ion indielectric crystals. J. Phys. Chem. Solids. 35, 897–899 (1974).

[b13] DeLosh R. G., Tien T. Y., Gibbons E. F., Zacmanidis P. J. & Stadler H. L. Strong Quenching of Tb^3+^ Emission by Tb–V Interaction in YPO_4_–YVO_4_. J. Chem. Phys. 53, 681–685 (1970).

[b14] Blasse G. & Bril A. Some considerations and experiments on concentration quenching of characteristic broad-band fluorescence. Philips Res. Rept. 23, 344–361 (1968).

[b15] Blasse G. & Bril A. Characteristic luminescence. Philips Tech. Rev. 31, 303–332 (1970).

[b16] Rai M., Mishra K., Singh S. K., Verma R. K. & Rai S. B. Infrared to visible upconversion in Ho^3+^/Yb^3+^ co-doped Y_2_O_3_ phosphor: Effect of laser input power and external temperature. Spectrochim. Acta A 97, 825–829 (2012). 10.1016/j.saa.2012.07.071 22902580

[b17] Luo X. & Cao W. Upconversion luminescence of holmium and ytterbium co-doped yttrium oxysulfide phosphor. Mater. Lett. 61, 3696–3700 (2007).

[b18] Vetrone F. . Temperature sensing using fluorescent nanothermometers. ACS Nano 4, 3254–3258 (2010). 20441184 10.1021/nn100244a

[b19] Brites C. D. S. . Thermometry at the nanoscale. Nanoscale 4, 4799–4829 (2012). 22763389 10.1039/c2nr30663h

[b20] Mahata M. K., Tiwari S. P., Mukherjee S., Kumar K. & Rai V. K. YVO_4_:Er^3+^/Yb^3+^ phosphor for multifunctional applications. J. Opt. Soc. Am. B 31, 1814–1821 (2014).

[b21] Saidi E. . Scanning thermal imaging by near-field fluorescence spectroscopy. Nanotechnology 20, 115703 (2009). 19420451 10.1088/0957-4484/20/11/115703

[b22] Henry D. M., Herringer J. H. & Djeu N. Response of 1.6 mum Er: Y_3_Al_5_O_12_ fiber-optic temperature sensor up to 1520 K. Appl. Phys. Lett. 74, 3447 (1999).

[b23] Wang S. P., Westcott S. & Chen W. Nanoparticle luminescence thermometry. J. Phys. Chem. B 106, 11203–11209 (2002).

[b24] Gavrilovic T. V., Jovanovic D. J., Lojpur V. & Dramicanin M. D. Multifunctional Eu^3+^- and Er^3+^/Yb^3+^-doped GdVO_4_ nanoparticles synthesized by reverse micelle method. Sci. Rep. 4, 4209 (2014). 24572638 10.1038/srep04209 PMC3936229

[b25] Fischer L. H., Harms G. S. & Wolfbeis O. S. Upconverting nanoparticles for nanoscale thermometry. Angew. Chem. Int. Ed. 50, 4546–4551 (2011). 10.1002/anie.201006835 21495125

[b26] Debasu M. L. . All‐in‐one optical heater‐thermometer nanoplatform operative from 300 to 2000 K based on Er^3+^ emission and blackbody radiation. Adv. Mater. 25, 4868–4874 (2013). 23696297 10.1002/adma.201300892

[b27] Mahata M. K., Kumar K. & Rai V. K. Er^3+^–Yb^3+^ doped vanadate nanocrystals: a highly sensitive thermographic phosphor and its optical nanoheater behavior. Sens. Actuators B 209, 775–780 (2015).

[b28] Dong B. . Optical thermometry through infrared excited green upconversion emissions in Er^3+^–Yb^3+^ codoped Al_2_O_3_. Appl. Phys. Lett. 90, 181117 (2007).

[b29] Lojpur V., Antić Ž. & Dramićanin M. D. Temperature sensing from the emission rise times of Eu^3+^ in SrY_2_O_4_. Phys. Chem. Chem. Phys. 16, 25636–25641 (2014). 25352320 10.1039/c4cp04141k

[b30] Ranson R. M., Evangelou E. & Thomas C. B. Modeling the fluorescent lifetime of Y_2_O_3_:Eu. Appl. Phys. Lett. 72, 2663 (1998).

[b31] Li X. . The emission rise time of BaY_2_ZnO_5_:Eu^3+^ for non-contact luminescence thermometry. J. Alloys Comp. 657, 353–357 (2016).

[b32] Goodenough J. B. Magnetism and the Chemical Bond (Interscience Publishers, 1963).

[b33] Huignard A., Buissette V., Laurent G., Gacoin T. & Boilot T. Emission processes in YVO_4_:Eu nanoparticles. J. Phys. Chem. B 107, 6754–6759 (2003).

[b34] Riwotzki K. & Haase M. Wet-chemical synthesis of doped colloidal nanoparticles: YVO_4_: Ln (Ln = Eu, Sm, Dy). J. Phys. Chem. B. 102, 1012910135 (1998).

[b35] Pollnau M., Gamelin D. R., Luethi S. R. & Guedel H. U. Power dependence of upconversion luminescence in lanthanide and transition-metal-ion systems. Phys. Rev. B 61, 3337 (2000).

[b36] Rodríguez V. D., Tikhomirov V. K., Velázquez J. J., Shestakov M. V. & Moshchalkov V. V. Visible-to-UV/Violet upconversion dynamics in Er^3+^-doped oxyfluoride nanoscale glass ceramics. Adv. Optical Mater. 1, 747–752 (2013).

[b37] Venikouas G. E. & Powell R. C. Laser time-resolved spectroscopy: Investigations of energy transfer in Eu^3+^ and Er^3+^ doped YVO_4_. J. Lumin. 16, 29–45 (1978).

[b38] Draai W. T. & Blasse G. Energy transfer from vanadate to rare‐earth ions in calcium sulphate. Phys. Stat. Sol. (a) 21, 569–579 (1974).

[b39] Riwotzki K. & Haase M. Colloidal YVO_4_: Eu and YP_0.95_V_0.05_O_4_: Eu nanoparticles: luminescence and energy transfer processes. J. Phys. Chem. B. 105, 12709–12713 (2001).

[b40] Wolf H. C. Energy transfer in organic molecular crystals: a survey of experiments. Adv. At. Mol. Opt. Phy. 3, 119–142 (1968).

[b41] Lojpur V., Ćulubrk S., Medić M. & Dramicanin M. Luminescence thermometry with Eu^3+^ doped GdAlO_3_. J. Lumin. 170, 467–471 (2016).

[b42] Marciniak L., Strek W. S., Hreniak D. & Guyot Y. Temperature of broadband anti-Stokes white emission in LiYbP_4_O_12_: Er nanocrystals. Appl. Phys. Lett. 105, 173113 (2014).

[b43] Mahata M. K. . Incorporation of Zn^2+^ ions into BaTiO_3_: Er^3+^/Yb^3+^ nanophosphor: an effective way to enhance upconversion, defect luminescence and temperature sensing. Phys. Chem. Chem. Phys. 17, 20741–20753 (2015). 26206553 10.1039/c5cp01874a

[b44] Sinha S., Mahata M. K. & Kumar K. Up/Down-Converted Green Luminescence of Er^3+^-Yb^3+^ Doped Paramagnetic Gadolinium Molybdate: a Highly Sensitive Thermographic Phosphor for Multifunctional Application. RSC Adv. 6, 89642–89654 (2016).

[b45] Lojpur V., Nikolić G. & Dramićanin M. D. Luminescence thermometry below room temperature via up-conversion emission of Y_2_O_3_: Yb^3+^, Er^3+^ nanophosphors. J. Appl. Phys. 115, 203106 (2014).

